# Foreign-trained dentists' reflections on access to care after participating in a community-based dental education curriculum

**DOI:** 10.3389/froh.2022.996624

**Published:** 2022-09-16

**Authors:** Patrick Dean Smith, Khatija Noorullah, Syeda Syed, Laila Iqbal, Scott L. Tomar

**Affiliations:** ^1^ Department of Pediatric Dentistry, Division of Prevention and Public Health Sciences, University of Illinois Chicago College of Dentistry, Chicago, IL, United States; ^2^ University of Illinois Chicago, Chicago, IL, United States

**Keywords:** community-based dental education, service-learning, foreign-trained dentists, advanced standing dental education, oral health, dental care access, underserved populations, community health

## Abstract

Many people suffer poor oral health due to dentists not providing care to them. The number of foreign-trained dentists in the US is increasing, yet little is known about their involvement in providing care to underserved populations. Dental education programs use community-based dental education (CBDE) to expose dental students to access to care issues, and encourage them to provide care to underserved populations upon graduation. The aim of this study was to assess foreign-trained dentists' attitudes about access to care issues after completing a CBDE course at a dental school in the Midwest. Fifty-two dentists participated in the CBDE program from 2018 to 2019, as part of an advanced standing curriculum, and completed guided, reflective essays. Forty-seven dentists agreed to have their essays anonymously coded for research. Four researchers reviewed the essays independently, developed a coding scheme, and recoded to agreement. The main themes dentists mentioned were the affect of the CBDE program on enhancing their clinical skills, fostering an awareness of healthcare system inadequacies, as well as an awareness of how specific social determinants limit access to care, and helping to encourage a sense of personal and professional responsibility to address access to care issues. This study highlights the value of CBDE on helping future dental providers learn about and reflect on access to care issues. It also provides insight into foreign-trained dentists' attitudes about access to care issues, and supports their participation in CBDE programs to foster their contributions in addressing access to care issues in the US.

## Introduction

In the US, there is a workforce shortage of dentists available to treat underserved populations. To help address those shortages, there has been an increase of 11 new dental schools since 2008 (1). There are also increasing numbers of foreign-trained dentists entering the oral health workforce in the US. Between 2011 and 2016, the number of H-1B visa applications, which allow U.S. companies to temporarily employ foreign-trained dentists, increased from 2.2 to 5.9 per 1000 population (2). The Health Resources and Services Administration estimates that as of June 2022, there are approximately 7,000 dental health professional shortage areas (D-HPSAs), with a gap of 11,673 dentists needed to remove D-HPSA designations (3). As the total number of dentists in the population increases, the extent that foreign-trained dentists can help to improve workforce shortages among underserved populations is unknown. From a policy perspective, foreign-trained dentists, unlike physicians, are not incentivized to work in underserved areas through J-1 visa waivers. As a result, the influence of foreign-trained dentists to work in D-HPSAs is limited to individual motivations. It is well known that most dentists, both US and foreign-trained choose not to treat underserved populations as part of their day to day careers. Most foreign-trained dentists pursue careers in private practice (4). Additionally, one study of dentists in Washington State revealed that foreign-trained dentists were significantly less likely than U.S.-trained dentists to accept Medicaid, and no more likely to practice in D-HPSAs than non-foreign trained dentists (5).

As a condition for licensure, many foreign-trained dentists enroll in Advanced Standing (AS) dental education programs to ensure that their training and practice philosophies align with U.S. standards (6). To date, there are 40 AS programs (7). The 2021–2022 American Dental Association (ADA) Survey of Dental Education reported that 704 graduates of international dental schools were admitted to U.S. dental schools, and non-resident aliens comprised 6.4% of all dental school graduates (8). Those numbers are projected to increase through 2037 (9).

The extent and level of training that foreign-trained dentists enrolled in AS dental education programs receive to work with underserved populations is unknown. AS dental education programs are typically shorter in length than conventional U.S. predoctoral dental programs, with a heightened focus on clinical competency (10). Additionally, special curricular modifications may be necessary to address AS participants' cultural norms, values, and beliefs; which may place demands on the available time that AS program participants have for learning about access to care issues in the US (11). Community-based dental education (CBDE) is an integral component of dental school curricula that is geared towards preparing dentists to treat underserved populations, and is a universal standard for predoctoral education (12). According to the Commission on Dental Accreditation (CODA) Standard 2–26, “Dental education programs must make available opportunities and encourage students to engage in service-learning experiences and/or community-based learning experiences [(13), p. 31].” In the academic year 2018–2019, 90.1% of U.S. dental schools required predoctoral students to participate in service-learning experiences, and 89.4% of dental schools required participation in community-based patient care experiences (14).

As a result of CBDE experiences, students have expressed more comfort and willingness to treat underserved populations (15–17). It has been reported that students who had CBDE experiences at multiple types of community clinics were more likely to choose community clinics as their first career choice (18, 19). Community partners of CBDE programs have expressed the benefits of being able to improve access to care through students' direct clinical care provision, and the opportunity to recruit dental students as future employees (20). Another hallmark of CBDE programs is the use of reflection to encourage students' critical thinking about access to care issues and assist in their professional development (21). Analysis of students’ reflections on CBDE experiences have revealed that students were more self-aware and empathetic towards patients’ needs, while challenging their preconceived stereotypes and assumptions about underserved populations (22–24). Students have also expressed an enhanced awareness of how healthcare relates to social justice issues (25).

CBDE highlights the complexity of social, political, and environmental influences on oral health and dental care delivery to underserved populations. It exposes students to models of care, populations, and communities that they otherwise might not have considered in their career plans. The reflective components built into the structure of CBDE curricula serve as a way to get students to self-assess and evaluate how their awareness and community-based experiences merge and align with their career goals. Hence, it is important that dental education programs facilitate all dental students' awareness of the oral health needs of patients who access community-based clinics for dental care.

There is limited knowledge of how AS dental education programs prepare foreign-trained dentists to practice in underserved communities, and how that preparation affects their attitudes about access to care issues in the US (5). It can be assumed that the shortened length and clinical focus of AS programs may limit the amount of curriculum time available for foreign-trained dentists enrolled in AS programs to have extensive experiences providing care in community-based settings. The objective of this study was to have a sample of foreign-trained dentists enrolled at one dental school complete a CBDE course and describe their attitudes about access to care issues in the U.S. The hope was that these data could inform how CBDE should be integrated and prioritized within the AS program curricula at one dental school in the Midwest.

## Materials and methods

This observational study was approved by the University of Illinois Chicago Institutional Review Board (Protocol # 2017-0767). As a pilot initiative, foreign trained dentists at one dental school in the Midwest were enrolled in a CBDE course during the final year of their AS program; May of 2018 to April of 2019. Participants completed online, asynchronous readings and written assignments, and at least one four-week clinical rotation. They also participated in four in-person, reflective class discussions. The readings, written assignments, and class discussions were divided into four learning modules covering: access to care; social determinants of health; interprofessional collaborative practice; and social and professional responsibility. The final written assignment was analyzed for this study. Participants were required to write a reflection essay discussing the following questions: How have you changed as a result of these experiences? What did you learn about the community, people, social justice, or other value systems? What did you learn about the health care system and health policy? What did you learn about how oral health fits within the health care system? A secondary intention of the reflection questions was to assess whether participants would discern the effect of social determinants of health on oral health and oral healthcare delivery. For clinical rotations, participants provided dental care to patients in community-based clinics under the direct supervision of adjunct faculty. Each faculty member was calibrated to understand their role in helping participants meet learning objectives of the CBDE course.

Questions to evaluate the CBDE course were included in the AS graduation exit survey. Eight questions assessed participants' age, gender, student loan debt, prior number of years in practice, and practice plans after graduation. Four questions asked students about the adequacy of their training, comfort, and preparedness to practice in community-based settings. The response categories for whether participants agreed or disagreed with having had adequate exposure to underserved populations through the CBDE course and whether they felt comfortable working with underserved populations were “agree”, “neutral,” and “disagree.” Categories of “very prepared,” “moderately prepared,” “somewhat prepared,” “minimally prepared,” and “not prepared” were used to assess the extent that students felt prepared for dental practice as a result of the CBDE course. Other categories of “significantly,” “moderately,” “somewhat,” “minimally,” and “not at all” were used to assess the extent that the CBDE course positively influenced participants' plans to work with underserved populations.

Before beginning the survey and final written assignment, each participant was given a consent document informing them that their responses could be used for research purposes, that minimal risk was involved, and that their responses would remain anonymous. Agreement to participate in the survey served as consent, and a written signature was obtained for qualitative analysis of participants' assignments. The survey was administered electronically *via* Qualtrics (Provo, UT, USA), and was analyzed by using SPSS (Chicago, IL, USA). Descriptive frequencies for each variable was calculated.

Prior to beginning the qualitative analysis, a member of the research team not affiliated with the CBDE course removed personal identifiers from each written assignment and assigned a unique identification number to each one. A team of four researchers conducted the qualitative analysis using thematic analysis (26). Coding was done using Google Sheets, a free web-based spreadsheet application that allowed researchers to code simultaneously in real time. Coding began with review and discussion of a random selection of several assignments to identify common themes and develop an initial coding scheme. Then, each researcher independently coded additional assignments, followed by team review and discussion of each written assignment until a general consensus about coding was reached. During that review process, themes were defined and refined. Three researchers repeated that process until each assignment was coded. Direct quotes were copied from assignments to ensure that the data accurately represented the themes that emerged.

## Results

Survey results are summarized in Table 1. Forty of 52 foreign-trained dentists enrolled in the CBDE course completed the survey, for a response rate of 76.9%. The mean age was 34 years (SD = 3.89). Students were 75% female and 25% male, with 85% having more than $300 K in educational debt. Twenty-three percent of responding participants had no dental practice experience before enrolling in the AS program and just over 50% had 1–3 years of practice experience. Only one respondent did not complete a CBDE clinical rotation. Nearly 65% of respondents had primary plans to enter into private practice immediately upon graduation, followed by additional training/residency programs (27.5%), and community-based organizations and government agencies (27.5%).

**Table 1:** Frequency and percent distributions of foreign-trained dentists’ responses to items from an assessment of a CBDE program that occurred May of 2018 to April of 2019, *N* = 40.

	*n* (%)
Mean Age = 34 y (SD = 3.89)	
Gender	
Male	10 (25.0)
Female	30 (75.0)
Years in practice prior to enrollment	
0 (Did not practice)	9 (22.5)
1–3	21 (52.5)
4–6	7 (17.5)
7 or more	3 (7.5)
Student loan debt	
$0–300,000	6 (15.0)
More than $300,000	34 (85.0)
Number of rotation blocks (1 block = 4 weeks)	
No rotation	1 (2.5)
One	35 (87.5)
More than one	4 (10.0)
Primary practice plans immediately after graduation (multiple response item)	
No plans	4 (10.0)
Government agency	4 (10.0)
Community-based organization	7 (17.5)
More training/residency	11 (27.5)
Private practice	24 (64.0)
Future vision of practice setting (multiple response item)	
Other (mobile dentistry)	1 (2.5)
Healthcare administration	8 (20.0)
Academic	14 (35.0)
Public health clinic (community-based/government)	18 (45.0)
Private practice	34 (85.0)
I envision devoting at least a portion of my time to uncompensated and/or voluntary dental care	
Agree	33 (82.5)
Neutral	7 (17.5)
Disagree	0 (0.0)
I had adequate exposure treating patients in community-based settings	
Agree	30 (75.0)
Neutral	9 (22.5)
Disagree	1 (2.5)
I am comfortable treating patients in a community-based setting	
Agree	36 (90.0)
Neutral	4 (10.0)
Disagree	0 (0.0)
Extent you feel prepared for practice after community-based rotations	
Very prepared	16 (40.0)
Moderately/Somewhat prepared	18 (45.0)
Minimally/Not prepared	6 (15.0)
Extent that community-based rotations positively influenced your plans to work with underserved populations	
Significantly	16 (40.0)
Moderately/Somewhat	19 (47.5)
Minimally/Not at all	5 (12.5)

The survey questioned participants about their level of exposure and comfort treating patients in community-based settings. It also asked them to describe the extent to which the CBDE course prepared them to enter into practice, and the extent to which that preparation positively influenced their plans to treat patients in underserved communities. When asked their agreement with having adequate exposure/training treating patients in public health/community-based clinical settings, 75% of responding participants agreed. Ninety percent agreed to being comfortable treating patients in community-based settings. The majority of respondents also reported that community-based rotations helped to prepare them for practice (85%), with about half of them reporting feeling only moderately or somewhat prepared. Similar findings were reported for responses to how community-based rotations positively influenced plans to work with underserved populations in respondents' dental careers.

Forty-seven written assignments were included in the qualitative analysis. Five main themes emerged from CBDE participants' reflections.

### Enhanced clinical knowledge and experience

Several participants appreciated that community-based rotations allowed them to experience dentistry in a real world setting compared to in the dental schools' clinics. Clinical benefits mentioned included working with trained dental assistants and becoming comfortable treating patients with special needs and patients from disadvantaged backgrounds. Participants also commented on their heightened confidence levels from the ability to treat more patients in a clinic session than they normally would at the dental school, and their improved speed and time management. “*After my rotation, I am more confident working independently, [and have] become more efficient and comfortable [with] time management*,” wrote one student. Other participants wrote about how the community-based clinical environment helped to improve their diagnostic and critical thinking skills. One participant mentioned, “*at the clinic, there was more flexibility, which allowed us to put into practice what we’ve learned in school. I learned to critique and evaluate my own work, then make decisions that would be for the good of my patient*.” Although the types of clinical skills that participants reported varied, the rotations appeared to benefit participants' overall professional development and clinical preparation to enter into practice upon graduation.

### Heightened awareness of how the healthcare system works for underserved populations

For this theme, participants reflected on how the needs of their patients were not being met, as a result of healthcare system issues. The context of many of the remarks for this theme was rooted in social justice. It was acknowledged by participants that, the “*health care system sometimes can be unfair to those who cannot afford it,” and “it is very hard for people with public insurance to find a place where their insurance is accepted*.” Some of those revelations were new, and others were drawn from comparisons with participants’ experiences in their home countries. For example, one participant commented *how “being an international student, and having seen the healthcare system in different countries, I realized that people with limited finances and insurance issues are the ones who suffer the most.*”

Several of the participants' reflections pointed towards the U.S. government as a source of blame and/or resources to improve access to care. One participant wrote, “*I remember how many times I asked myself why the government did not do anything to fix that issue,”* and another noted, “*there needs to be more funding and support from the government to help us [dentists] help people.*” Participants remarked on the value of Medicaid, while also acknowledging that many providers are unwilling to treat Medicaid recipients. Knowledge of the existence of community health centers to improving access to care was also mentioned. As stated by one participant, “*it was new to me that there are places in our community, made by our community, to help the community without any profit*.” Participants' written assignments revealed that they were thinking about access to care issues of insurance and affordability, and the availability of government and community resources to address those issues.

### Barriers patients face seeking dental care

Another theme that emerged from the written assignment was participants gaining an awareness of the barriers that some people face in accessing dental care. Several participants commented on the financial barriers of receiving dental care and how dentistry “*is a luxury that they [people] cannot afford*,” causing many people to only access dental care when they were in pain. One participant commented on how such treatment delays can contribute to more dental needs among people with access to care barriers by writing*, “a lot of the procedures performed on these patients could have been avoided if the patient was treated earlier.*” Another barrier that was mentioned was transportation, with one participant stating that, “*people in the [community clinic] were coming from as far as 100 miles to get their tooth fixed.*” Another participant discussed the importance of public transportation, as most of the patients at his/her rotation site relied upon it to get to dental appointments. Other participants mentioned gaps in oral health education and awareness, which can hinder patients' willingness to accept dental care. This was highlighted by one participant who wrote, “*parents don’t always prioritize [their children's] oral health; not because they don’t care, but because they don’t have the information they need*.” Some participants also commented on their beliefs that some patients were not taking dental care seriously or did not value their teeth, as evidenced by high numbers of canceled dental visits and by patients only seeking emergency dental care. Overall, results revealed that participants were able to identify and reflect on internal and external barriers to dental care, such as patients' levels of knowledge, attitudes, transportation, and/or their ability to afford care. Participants were also able to witness how those barriers may negatively affect people's oral health.

### Professional responsibility

This theme was discussed in the assignments from two main points of view. Some participants mentioned the responsibility of dentists as a collective body, while others wrote about how their experience in the course shaped their personal obligations to address access to care among their patients. For example, one participant wrote, “*after my experience at [xxx], I felt the need to do something for underserved populations*,” and another wrote, “*I started thinking about how I could contribute to people who could otherwise not afford treatment*.” In discussing professional responsibility as a collective body of dental professionals, one participant mentioned, “*every dentist must listen to their social conscience and the often-unheard voices of the neglected children, the elderly, the disabled, and the culturally marginalized members of society*.” Another wrote, “*it's our responsibility as dentists and as [a] community to take action in trying to improve oral health and raise awareness for many still remaining problems*.” These responses suggest that the CBDE course played a role in helping participants think about their role and the profession's role to do more to address issues related to access to care.

### Personal satisfaction of helping people

Participants acknowledged their own positive feelings from witnessing how patients benefited from the care that they provided. One participant wrote, “*it was a great feeling to help people and alleviate their pain and make them feel like they had an option and a place to go for dental treatment*.” Other participants introduced this theme in their writing by reflecting on how the experience enhanced their perspectives and even helped to shape their careers. According to one participant, “*this experience has changed my perception and now I would like to work and serve [in an] underserved population. I am planning to start looking for a job or plan to have a dental van for such [a] population*.” For those participants who made similar remarks, the value of CBDE for them also appeared to be its ability to offer them a general sense of fulfillment and desire to use their skills to serve others.

## Discussion

This assessment provided insight into what foreign-trained dentists can learn about access to care issues and themselves from participating in CBDE programs. In general, participants' reflections about their enhanced clinical experiences were positive and consistent with what has been reported in previous studies (27–31). Participants expressed an awareness of issues related to access to care that directly impacts patients and issues that demand more systematic approaches. Behar-Horenstein and colleagues reported a similar finding in their study of predental students (25). The issues that participants mentioned also were consistent with systematic issues that the Health Policy Institute of the American Dental Association has identified, such as a need for improvements in insurance coverage, reimbursement systems, and care delivery systems to benefit populations most in need (32).

Another result that stands out in this study is the difference in the percentage of survey respondents who felt that they had adequate exposure to treating patients in community-based settings and the percentage that expressed comfort working in those settings. That difference could be due to a couple of scenarios. Respondents may have simply wanted more weeks of clinical rotation experience. They could also have been more comfortable working in such settings based on previous dental practice experiences. It has been reported that community-based rotations that are eight weeks or longer in duration are more meaningful than shorter rotations for students (33), so a rotation longer than the four weeks might increase the percentage of respondents feeling as if their exposure and training in community-based sites was adequate. Additionally, Thind and colleagues reported that higher social consciousness attitudes were significantly associated with improving dental students' ability to provide care to racially, ethnically, and culturally diverse groups (34). Foreign-trained dentists may be prone to having positive educational experiences with underserved populations due to their maturity and previous clinical experiences. They may also have higher levels of cultural competency and social consciousness by way of their unique and diverse life experiences (6, 11, 35).

Participants in this CBDE program also shared reflections on their personal satisfaction of helping people, and personal and professional responsibility to address access to care issues. Results from other studies regarding this sentiment among dental students have been mixed. In some studies, dental students have reported decreased idealism about their desires to treat underserved populations or to volunteer as they progressed through dental school (36, 37). At least one report revealed that students participating in CBDE were more likely to provide charitable care as practicing dentists (38).

More research is needed to determine how the benefits of CBDE for foreign-trained dentists may play out in their professional careers. Will they become more motivated to become Medicaid providers or practice in health professional shortage areas? Other factors influencing career choices of foreign-trained dentists, such as educational indebtedness, practice location, and cultural and social influences must be considered and studied. Future work in this domain could also assess how those influences of practice preference factor into foreign-trained dentists' willingness and intent to treat underserved populations or work in community-based settings. Additionally, AS dental education programs can assess potential barriers that exist for foreign-trained dentists enrolled in their programs to have adequate and meaningful community-based experiences embedded into curricula. A possibility for a future study is to investigate any differences in attitudes towards underserved populations between foreign-trained dentists and predoctoral dental students, and what factors may contribute to any differences, such as age, gender, race, professional experience, and socio-cultural influences that may not be common among most traditional predoctoral dental students. For example, women and dentists from historically underrepresented race and ethnic groups are more likely to work in underserved communities, and may also have a high likelihood of being foreign-trained (39, 40). This is important in the context of the the culturally diverse demographics of foreign-trained dentists. Having such context may allow more in depth analysis of what student characteristics are more closely associated with the influence of CBDE on dentists' willingness to work with underserved populations.

Knowledge generated from this study's results provide new knowledge on the value of CBDE to foreign-trained dentists' education; supporting the existence of curricula geared towards their preparation to practice with underserved populations. The extent that foreign-trained dentists enrolled in AS programs at other dental schools participate in CBDE is not known. However, this study supports the feasibility of having AS students participate in CBDE, which includes at least four weeks of clinical rotation supported by didactic content and reflective self-assessments.

### Strengths and limitations of the study

This study reports on the feasibility and value of CBDE in educating foreign-trained dentists about access to care issues in the U.S. and preparing them for community-based practice. To the best of our knowledge, this has not been reported in pre-existing literature. However, it should be noted that the focus of CBDE is on individual experiences that are only part of a solution to a highly complex issue. For example, participants in this study were able to articulate health system challenges that were beyond their clinical and CBDE experiences, such as how to address patient level barriers and advocate for improved public insurance coverage and reimbursement. Also, participants in this study did not mention gender, race and/or ethnicity as health system and patient-level issues that need to be addressed. The potential for CBDE to have a greater impact on students' knowledge and attitudes about health system issues lies in the ability of CBDE to delve beyond clinical rotations and provide more training on how to design and implement clinical systems and policy changes with greater potential for impact (21).

Another strength of this study is that although all participants received the same didactic content, not all of them rotated to the same community-based sites. Therefore, participants' reflections were not contingent on them experiencing the same learning environments. This highlights the similarity of access to care challenges among various locations and population groups, and participants' individual assessment of those challenges. One of the limitations of this study is that this was a sample of one cohort of foreign-trained dentists enrolled in an AS program at one dental school, so results cannot be generalized. There was possible response bias as not all foreign-trained dentists enrolled in the AS program participated in the survey or consented to use their written assignments for research. Thus, those foreign-trained dentists who chose not to participate may have had more negative experiences and reflections than those who did participate. It is also possible that participants embellished their survey responses and reflections to appeal to the CBDE course faculty, rather than stating their true attitudes and reflections. Finally, participants' attitudes about underserved populations and community-based dental practice may have existed prior to their participation in this CBDE course. In repeating this study, AS students could be given a pre-survey to evaluate the extent that the CBDE course changes their levels of comfort and feelings of preparedness. Students can also participate in more than one clinical rotation to assess the effect of more rotation weeks. Finally, data can be collected to assess the impact of various course content and experiences on shaping foreign-trained dentists' attitudes about access to care issues.

## Contribution to the field

This study revealed that CBDE can raise the level of awareness that foreign-trained dentists have about access to care issues among underserved populations in the US, and nurture their interest in addressing access to care issues in their careers. Participants in this study were able to identify health system and patient-level barriers to dental care, while also helping to instill a sense of responsibility and satisfaction in their capacity to address oral health among underserved populations. These findings support CBDE in AS program curricula to prepare foreign-trained dentists to address the oral health needs of underserved populations in the U.S.

## Data Availability

The original contributions presented in the study are included in the article/supplementary materials, further inquiries can be directed to the corresponding author/s.
